# Intestinal ischemia–reperfusion and blood–brain barrier compromise: pathways to cognitive dysfunction

**DOI:** 10.3389/fnins.2025.1597170

**Published:** 2025-07-15

**Authors:** Opeyemi Hammed, Oladele Afolabi, Richard Ajike, Oluwaseun Hezekiah, Babatunde Alabi, David Ajao, Waidi Saka, Olubunmi Oyekunle, Bamidele Olusola

**Affiliations:** ^1^Department of Physiology, College of Health Sciences, Ladoke Akintola University of Technology, Ogbomoso, Nigeria; ^2^Experimental Animal Research Division, Helix Biogen Institute, Ogbomoso, Nigeria; ^3^PACRI, Faculty of Heath Sciences, University of Pretoria, Prinshof Campus, South Africa; ^4^Department of Nursing, Kings University, Ode-Omu, Nigeria; ^5^Department of Public Health, College of Health Sciences and Public Policy, Walden University, Minneapolis, TX, United States

**Keywords:** intestinal ischemia–reperfusion injury, blood–brain barrier, cognitive dysfunction, systemic inflammation, gut microbiota dysbiosis, gut microbial metabolites

## Abstract

Intestinal ischemia–reperfusion (I/R) injury, a disorder occurring from interruption of blood flow to the intestines followed by its restoration, causes a cascade of events leading to systemic consequences, including cognitive impairment. This study analyses the complicated link between intestinal I/R damage and blood–brain barrier (BBB) compromise, highlighting essential processes such as systemic inflammation, gut microbiota dysbiosis, oxidative stress, vagus nerve activation, and altered gut microbial metabolite production. During I/R injury, the weakened gut barrier permits the translocation of microbial products and inflammatory mediators into the circulation, beginning systemic inflammation that disrupts the BBB and exacerbates neuronal damage. Furthermore, gut microbiota dysbiosis and altered gut microbial metabolite synthesis, such as short-chain fatty acids (SCFAs), can impact neuronal signaling and cognitive processes. By delineating these pathways, this study seeks to provide a comprehensive knowledge of the intricate interplay between intestinal I/R injury, BBB integrity, and cognitive function, opening the way for potential therapeutic approaches.

## 1 Introduction

Intestinal ischemia–reperfusion (I/R) injury refers to the damage sustained by the intestinal tissue due to a transient lack of blood flow (ischemia) followed by the restoration of circulation (reperfusion) (Hummitzsch et al., 2019). This event commonly occurs in conditions such as trauma, surgery, or shock, leading to compromised intestinal integrity. The initial ischemic phase results in a scarcity of oxygen and nutrients, triggering cellular injury, oxidative stress, and inflammation (Archontakis-Barakakis et al., 2025). However, the subsequent reperfusion, meant to restore normal blood flow, exacerbates the damage through the formation of reactive oxygen species (ROS), inflammatory mediators, and the activation of numerous signaling pathways (Sánchez-Hernández et al., 2020). This double-edged character of I/R injury contributes to significant tissue damage, and it is commonly related with systemic consequences, including multi-organ failure (MOF) (Sánchez-Hernández et al., 2020). The blood–brain barrier (BBB) is a highly selective, a border between the blood circulation and the central nervous system (CNS), necessary for maintaining homeostasis and protecting the brain from hazardous substances (Daneman and Prat, 2015). The BBB consists of endothelial cells tightly joined by tight junctions, supported by astrocytic end-feet and pericytes, which collectively regulate the movement of ions, nutrients, and metabolic waste across the barrier while preventing the entry of pathogens, toxins, and potentially harmful immune cells (Abbott et al., 2010). In addition to its protective function, the BBB serves a crucial role in maintaining the brain’s microenvironment and controlling cerebral blood flow, which is essential for healthy cognitive functioning. Any disruption to BBB integrity can lead to altered cerebral function, contributing to different neurological diseases, including cognitive dysfunction (Ronaldson and Davis, 2020; Rajeev et al., 2022).

Emerging evidence suggests a bidirectional relationship between gut health and brain function, often referred to as the “gut-brain axis.” Intestinal I/R injury, as a form of acute gut stress, has been implicated in triggering systemic inflammatory responses that extend beyond the gut, to impact distant organs, including the brain (Osadchiy et al., 2019). During I/R injury, the intestinal barrier becomes weakened, allowing microbial translocation, endotoxins, and inflammatory mediators such as cytokines to enter the bloodstream. This leads to systemic inflammation, which might impact the permeability of the BBB and worsen neuronal injury (Zhang et al., 2024a). Moreover, gut microbiota dysbiosis, resulting from intestinal I/R injury, increases intestinal permeability, allowing bacteria and their products (e.g., lipopolysaccharide, LPS) to translocate into the circulation, activating systemic inflammation and disrupting the BBB. Furthermore, intestinal I/R injury can activate the vagus nerve, transmitting signals to the brain that influence neuronal activity and cognitive function, and alter gut microbial metabolite production, such as short-chain fatty acids (SCFAs), thus affecting neuronal signaling and cognitive processes (Wu et al., 2021; Zhang et al., 2024b).

Given these linkages, this review gives a complete summary of how intestinal I/R can lead to BBB malfunction and cognitive impairment. It also explores specific pathways, including the role of systemic inflammation in BBB disruption and neuroinflammation, the impact of gut microbiota dysbiosis on intestinal permeability and translocation of microbial products, the contribution of oxidative stress to BBB damage, the potential involvement of the vagus nerve in transmitting signals from the gut to the brain, and the effects of altered gut microbial metabolite production on neuronal function. By exploring these pathways, this study attempts to expand the understanding of the complex link between intestinal I/R injury, BBB integrity, and cognitive function, and possibly opening the way for novel therapeutic options.

## 2 Intestinal ischemia–reperfusion (I/R) injury: mechanisms of intestinal ischemia

Intestinal ischemia is a crucial clinical challenge defined by inadequate blood supply to the intestines, depriving said tissue of essential oxygen and nutrients. This deprivation triggers a complex cascade of cellular and molecular reactions that, if left unchecked, culminate in major tissue damage and potentially life-threatening issues. The severity of intestinal ischemia rests on the degree and duration of blood flow loss, influencing the level of cellular destruction and the risk for subsequent reperfusion injury (Dorweiler et al., 2007; Vollmar and Menger, 2011).

### 2.1 Reduced blood flow to the intestine: a multifaceted etiology

The causes of diminished intestinal blood flow are various, resulting from a number of occlusive and non-occlusive factors. Occlusive ischemia involves a physical obstruction within the mesenteric vasculature, either in the arteries or veins. Arterial occlusion often originates from thromboembolism, where blood clots originating elsewhere in the body lodge within the mesenteric arteries, blocking blood flow (Yu and Kirkpatrick, 2023). Atherosclerosis, which is usually characterized by slow plaque formation inside the artery walls, can also progressively constrict the mesenteric arteries, ultimately leading to critical stenosis and reduced perfusion. Rarer causes include arterial dissection and vasculitis, as well as inflammation of the blood vessel walls (Schramm et al., 2011). Mesenteric venous thrombosis, involving blood clot development within the mesenteric veins, constitutes another prominent cause of intestinal ischemia. This condition commonly arises in persons with underlying hypercoagulable conditions, such as genetic clotting abnormalities, cancer, or following recent surgical procedures (Stancu et al., 2024).

Non-occlusive mesenteric ischemia (NOMI), in disparity, does not include a physical occlusion of the mesenteric arteries. Instead, diminished blood flow arises from systemic causes that limit intestinal perfusion. Low cardiac output, arising from situations such as heart failure, cardiogenic shock, or severe hypovolemia, can lead to insufficient blood transport to the intestines (Al-Diery et al., 2019). Additionally, systemic vasoconstrictors, like vasopressors used to treat hypotension and certain drugs like ergotamine, can restrict the mesenteric arteries, thereby limiting blood flow. Splanchnic vasoconstriction, produced by the production of vasoconstrictive hormones and neurotransmitters in response to stress, pain, or cold, can also contribute to NOMI (Hass et al., 2007). Even when large mesenteric arteries remain patent, poor microcirculation within the gut wall might contribute to ischemia damage. Endothelial dysfunction, characterized by damage to the cells lining the micro vessels, reduces their ability to regulate blood flow and permeability (Sukriti et al., 2014). The creation of microthrombi inside the microcirculation can also hinder blood flow, while increasing blood viscosity (thickness) can impede blood flow via these smaller capillaries (Miranda et al., 2016).

### 2.2 Cellular injury and necrosis during ischemia: a pathophysiological cascade

The decline in blood flow during ischemia triggers a cascade of biological events that ultimately lead to cell death. Oxygen deprivation (hypoxia) represents the immediate result, since intestinal cells are deprived of the oxygen necessary for mitochondrial oxidative phosphorylation, the major process for creating ATP (Weinberg et al., 2000; Fan et al., 2013). The ensuing ATP depletion disturbs a variety of cellular operations requiring energy, including the activity of ATP-dependent ion pumps, such as the Na+/K + ATPase. The failure of these pumps leads to an imbalance of ions across the cell membrane, with an influx of sodium and calcium into the cell and an efflux of potassium (de Lores Arnaiz, 2007; Muangkram et al., 2023). Furthermore, ATP depletion affects cell membrane integrity, leading to instability and leakage. The influx of calcium into the cell stimulates calcium-dependent enzymes, including phospholipases, proteases, and endonucleases, which contribute to cellular damage by degrading membrane phospholipids, breaking down intracellular proteins, and fragmenting DNA, respectively (Khatri et al., 2021). As cellular metabolism turns to anaerobic glycolysis, lactic acid builds, resulting to intracellular acidosis, further compromising cellular function and contributing to cell damage (Li et al., 2022).

Ischemia can trigger both apoptotic and necrotic cell death pathways. Apoptosis, or planned cell death, is characterized by DNA fragmentation, cell shrinkage, and the development of apoptotic bodies (D’arcy, 2019). Necrosis, in divergence, represents uncontrolled cell death, characterized by cell enlargement, membrane rupture, and the release of intracellular contents, leading to inflammation (Bertheloot et al., 2021). Crucially, ischemia injury impairs the integrity of the intestinal epithelial barrier, resulting to increased permeability. This weakened barrier permits bacteria and bacterial products, such as lipopolysaccharide (LPS), to translocate across the intestinal wall and enter the bloodstream, contributing to systemic inflammation and sepsis (Ghosh et al., 2020). The cumulative impact of these cellular activities results in cell death and necrosis of the intestinal tissues. The level of tissue damage is dictated by the intensity and length of ischemia, as well as the individual’s overall health and any underlying diseases (Vollmar and Menger, 2011).

### 2.3 Reperfusion and related inflammatory response

The reperfusion phase following intestinal ischemia is defined by the return of blood flow to the ischemic tissue. While this is designed to restore oxygen and nutrition flow, it paradoxically exacerbates the injury suffered during ischemia (Eltzschig and Eckle, 2011). This condition, known as ischemia–reperfusion (I/R) injury, is distinguished by the formation of reactive oxygen species (ROS), the activation of inflammatory mediators, and extensive tissue inflammation (Soares et al., 2019). These responses play a key role in tissue destruction, particularly in the intestinal mucosa, and contribute to systemic consequences, including multi-organ failure. The inflammatory responses induced during reperfusion are crucial to the development of I/R injury and associated consequences, including blood–brain barrier (BBB) impairment and cognitive dysfunction (Venkat et al., 2017).

#### 2.3.1 Reactive oxygen species (ROS) generation

During the reperfusion phase, the restoration of oxygen to previously ischemic tissue leads to a quick and overwhelming generation of ROS. ROS are extremely reactive chemicals that comprise free radicals (such as superoxide anion, O_2_^−^), non-radical species (such hydrogen peroxide, H_2_O_2_), and hydroxyl radicals (OH) (Bugger and Pfeil, 2020). Under normal settings, ROS are created as byproducts of cellular respiration and serve crucial roles in signaling cascades and immune defense. However, during reperfusion, the quick input of oxygen creates an overproduction of ROS, which can overwhelm the body’s antioxidant defense systems, resulting in oxidative stress (Liu et al., 2018). This spike in ROS levels has various negative impacts on tissue. ROS can directly damage biological structures, including lipids, proteins, and DNA, contributing to cell death, necrosis, and apoptosis (Juan et al., 2021). In the case of intestinal I/R injury, ROS cause the oxidation of lipids inside cellular membranes, leading to lipid peroxidation, which destabilizes membrane integrity and increases cellular permeability. This stimulates the leakage of intracellular contents and aids the recruitment of inflammatory cells, further continuing tissue injury (Calcerrada et al., 2011).

A major source of ROS during reperfusion is from the mitochondria, as a result of mitochondrial dysfunction. In the mitochondria, small amounts of ROS are generated as by-products of oxidative phosphorylation. These ROS are quickly neutralized by the body’s antioxidant defence before they can damage important cytoplasmic organelles (Schang et al., 2016). However, in pathological states, such as during reperfusion, the generation of ROS from the electron transport chain (ETC), particularly complexes I and III, exceeds the normal resulting in leakage of electrons that react with molecular oxygen to form superoxides. In addition, enzymes, such as xanthine oxidase, NADPH oxidase (NOX), and myeloperoxidase (MPO), become overtly hyperactive during reperfusion and significantly contribute to the production of ROS (Sasaki and Joh, 2007; Granger and Kvietys, 2015).

Moreover, ROS stimulate many signaling pathways, including the NF-κB pathway, which governs the expression of pro-inflammatory cytokines and chemokines. ROS also induce the release of matrix metalloproteinases (MMPs), enzymes that damage the extracellular matrix and affect tissue integrity (Bryan et al., 2012). The activation of these pathways sets the foundation for systemic inflammation, exacerbating the local injury and potentially harming distant organs such as the brain. In addition to their direct biological effects, ROS contribute to the opening of tight junctions in endothelial cells of the intestinal vasculature, leading to enhanced vascular permeability (Sukriti et al., 2014; Wautier and Wautier, 2022). This allows for the passage of germs, endotoxins, and other toxic chemicals into the bloodstream, which can induce a systemic inflammatory response and further worsen BBB failure (Gu et al., 2021).

#### 2.3.2 Inflammatory mediators (Cytokines, Chemokines)

The inflammatory response that accompanies I/R injury is orchestrated by a number of inflammatory mediators, including cytokines, chemokines, and adhesion molecules. These chemicals are generated from injured or stressed cells in response to ROS formation and operate as signaling factors to recruit and activate immune cells, sustaining inflammation (Albano et al., 2022). Cytokines are tiny proteins that govern the immune response by stimulating inflammation, tissue healing, and immune cell activation (Zhao et al., 2021). During reperfusion, the ischemic tissues release pro-inflammatory cytokines, such as tumor necrosis factor-alpha (TNF-*α*), interleukin-1β (IL-1β), and interleukin-6 (IL-6), which contribute to the recruitment of neutrophils, macrophages, and other immune cells to the site of injury (Pawluk et al., 2020; Jurcau and Simion, 2021). TNF-α and IL-1β, in particular, are major drivers of inflammation in response to I/R injury. These cytokines launch the inflammatory cascade by activating transcription factors like NF-κB, which subsequently upregulate the expression of other cytokines, chemokines, and adhesion molecules. The enhanced inflammatory response generates a vicious loop of immune cell recruitment, tissue injury, and additional cytokine production (Vendramini-Costa and Carvalho, 2012; Hunter and De Plaen, 2014).

Chemokines, another type of signaling molecules, are involved in the recruitment and activation of leukocytes during the inflammatory response (Turner et al., 2014). Chemokines such as monocyte chemoattractant protein-1 (MCP-1), regulated upon activation normal T cell expressed and secreted (RANTES), and macrophage inflammatory protein-2 (MIP-2) are released in response to I/R injury (Koo and Fuggle, 2002; Turner et al., 2014). These chemokines attach to specific receptors on the surface of immune cells, driving their migration to the site of injury (Tiberio et al., 2018). In the condition of intestinal I/R injury, the migration of neutrophils and monocytes into the gut tissue exacerbates local inflammation, leading to the production of more ROS, proteolytic enzymes, and pro-inflammatory mediators. This promotes additional breakdown of the intestinal barrier, exacerbating tissue injury and potentially leading to systemic infection (Arosa et al., 2022; Saez et al., 2023). Additionally, the release of intercellular adhesion molecules (ICAM-1) and vascular cell adhesion molecules (VCAM-1) on endothelial cells enhances the adherence and extravasation of leukocytes into the inflamed tissue (Habas and Shang, 2018). These adhesion molecules are crucial for the migration of immune cells across the blood artery wall, and their overexpression during reperfusion adds to tissue inflammation and the possibility for multi-organ failure (Anzai et al., 2022). The stimulation of these pathways not only impacts the intestinal tissue but can also have far-reaching effects on other organs, including the brain (Zhou et al., 2012).

The systemic inflammatory response generated by the production of cytokines and chemokines during intestinal I/R injury might profoundly disrupt the blood–brain barrier (BBB) (Guo et al., 2025). Pro-inflammatory cytokines such as TNF-*α*, IL-1β, and IL-6 can enhance BBB permeability by altering tight junction proteins, resulting to a loss of barrier integrity. This allows the influx of immune cells, toxins, and other inflammatory mediators into the brain, contributing to neuroinflammation and possibly cognitive impairment (Voirin et al., 2020; Gu et al., 2021).

## 3 Systemic consequences of intestinal ischemia–reperfusion injury

### 3.1 Increased gut permeability and endotoxemia

Intestinal ischemia–reperfusion damage is defined by a cascade of inflammatory and vascular processes that result in an increase in intestinal permeability. The impaired gut barrier function is a characteristic of I/R injury and has broad systemic effects, including the development of endotoxemia (Sharapov et al., 2017; Huang et al., 2024).

#### 3.1.1 Increased gut permeability

During ischemia, the absence of blood flow produces hypoxia and cellular injury within the intestinal epithelial cells, leading to disruption of tight junction proteins such as occludin, claudins, and zonula occludens (Abdullahi et al., 2018). These tight junctions generally protect the integrity of the intestinal epithelium by regulating the paracellular movement of solutes and bacteria. Ischemia-induced oxidative stress and inflammation worsen epithelial damage, which leads in an increased permeability of the intestinal lining, allowing the transfer of bacteria, their products, and toxins from the gut lumen into the circulation (Liao et al., 2022). Upon reperfusion, the restoration of blood flow exacerbates the inflammatory response through the formation of reactive oxygen species (ROS) and the activation of immune cells such as neutrophils and macrophages. These variables contribute further to the collapse of the epithelial barrier. Increased gut permeability after I/R damage is not just a localized issue; it has systemic repercussions, leading to endotoxemia (Canton et al., 2021; Ucar and Ucar, 2021).

#### 3.1.2 Endotoxemia

Endotoxemia refers to the presence of endotoxins, primarily lipopolysaccharides (LPS) from the outer membrane of Gram-negative bacteria, in the bloodstream. In a healthy state, the intestinal mucosa works as a selective barrier, preventing bacterial endotoxins from entering circulation (Nishizawa, 2016). However, following intestinal I/R injury, the injured intestinal epithelium becomes leaky, letting bacterial components such as LPS to reach the systemic circulation (Wang et al., 2019; Vanuytsel et al., 2021). LPS is a strong stimulator of the immune system. It binds to toll-like receptors (TLRs), specifically TLR4, which are expressed on numerous immune cells like macrophages, neutrophils, and dendritic cells (Wicherska-Pawłowska et al., 2021). The activation of TLR4 causes a cascade of inflammatory signaling pathways, including the nuclear factor kappa B (NF-κB) pathway, which leads to the release of pro-inflammatory cytokines such as tumor necrosis factor-alpha (TNF-*α*), interleukin-1 (IL-1), and interleukin-6 (IL-6). These cytokines contribute to systemic inflammation and play a role in the evolution of multi-organ dysfunction (Hunter and De Plaen, 2014; Gourd and Nikitas, 2020). The presence of endotoxins in the bloodstream can lead to various adverse effects, including sepsis, systemic inflammatory response syndrome (SIRS), and the worsening of already existing diseases like liver dysfunction, renal failure, and acute respiratory distress syndrome (ARDS). In I/R damage, endotoxemia may further disrupt the blood–brain barrier (BBB), contributing to neuroinflammation and cognitive impairment (Gourd and Nikitas, 2020).

### 3.2 Potential role of the gut-brain axis

The gut-brain axis refers to the bidirectional communication network between the gastrointestinal system and the central nervous system (CNS). It encompasses several pathways, including neurological (vagus nerve), hormonal, immunological, and microbiome-mediated signals, all of which play a critical role in maintaining physiological homeostasis and responding to systemic stresses (Sasso et al., 2023). Recent studies suggest that intestinal I/R injury may alter this delicate equilibrium, facilitating the passage of peripheral inflammatory signals to the brain, leading to neuroinflammation, and eventually compromising cognitive performance (Song et al., 2020; Jurcau and Simion, 2021).

#### 3.2.1 Gut-Bacterial microbiome and inflammation

The gut microbiome, comprised of billions of microorganisms, has a key role in maintaining gut homeostasis. Under normal settings, the microbiota helps regulate immune responses, defend the intestinal barrier, and maintain local and systemic metabolic processes (Spiljar et al., 2017). However, intestinal I/R damage affects the gut microbial environment. The enhanced permeability generated by I/R damage facilitates translocation of not only bacterial products (such as LPS) but also live microorganisms and peptidoglycans, which stimulate systemic immune responses (Gao et al., 2023). These microbial-derived molecules act as pathogen-associated molecular patterns (PAMPs), activating pattern recognition receptors (PRRs) on peripheral immune cells and brain-resident microglia. Research has showed that alterations in the gut microbiome following I/R injury may induce an inflammatory cascade that alters brain function (Nadatani et al., 2018). The production of microbial metabolites and inflammatory mediators such as cytokines and chemokines can activate the brain’s innate immune cells, notably microglia. Chronic activation of microglia shifts it to a proinflammatory M1 phenotype, which releases ROS, nitric oxide, and additional cytokines. These events are consistent with neuroinflammation, a process that is related with different cognitive impairments, including memory loss and decreased learning capacity (Kaur et al., 2019).

### 3.3 Neuroinflammation and cognitive dysfunction

Neuroinflammation is a well-established process leading to cognitive failure in several neurological illnesses, including Alzheimer’s disease, Parkinson’s disease, and stroke (Skrzypczak-Wiercioch and Sałat, 2022). In the case of intestinal I/R injury, the systemic inflammatory response generated by endotoxemia and altered gut microbiota can activate the brain’s immune cells. Microglia, upon activation, emit pro-inflammatory cytokines and free radicals, which not only increase neuronal injury but also impair synaptic plasticity, a critical step for learning and memory (Bourgognon and Cavanagh, 2020). Furthermore, the vagus nerve plays a crucial role in gut-brain communication. The afferent fibers of the vagus nerve send information from the gut to the brain (Wang et al., 2024a). In response to intestinal inflammation and oxidative stress generated by I/R injury, the vagus nerve may be implicated in influencing the brain’s inflammatory response (Shahrokhi et al., 2016). Studies have revealed that vagal nerve stimulation can attenuate neuroinflammation and improve cognitive performance, underscoring the importance of this neural route in preserving brain health under conditions of peripheral injury (Daulatzai et al., 2014; Neren et al., 2016; Gargus et al., 2025).

### 3.4 Blood–brain barrier compromise

One of the most serious systemic outcomes of intestinal I/R injury is the possible disruption of the blood–brain barrier (BBB). The BBB serves as a selective barrier protecting the brain from circulating viruses and poisons. In the case of I/R injury, systemic inflammation and the release of cytokines and LPS can enhance BBB permeability by breaking tight junctions between endothelial cells and by activating matrix metalloproteinases that breakdown the extracellular matrix (Sá-Pereira et al., 2012). This leads in the release of potentially toxic compounds into the brain, further worsening neuroinflammation and leading to the development of cognitive impairment. Animal studies have demonstrated that intestinal I/R damage can lead to BBB malfunction and cognitive abnormalities such as deficiencies in memory and learning (Sá-Pereira et al., 2012). Increased BBB permeability permits the entry of inflammatory cytokines, LPS, and other neurotoxic chemicals that can induce or worsen neuroinflammatory responses, further affecting brain function. The ensuing neuroinflammation can emerge as long-term cognitive impairment and neurodegeneration (Haruwaka et al., 2019).

## 4 Blood–brain barrier (BBB) compromise due to systemic inflammation

### 4.1 Structure and function of the blood–brain barrier (BBB)

The blood–brain barrier (BBB) is an important structure in the central nervous system (CNS) that largely serves the purpose of selective permeability. The CNS environment is tightly regulated to prevent poisons and other undesired chemicals from seeping into the parenchymal area. This guarantees that the CNS environment is always tightly maintained to be able to serve its crucial job of regulating and managing other body functions (Neuwelt et al., 2011; Obermeier et al., 2016). On the structural level, this barrier is made up of different cells including pericytes, basement membrane, mural cells, some immune cells, and endothelial cells joined together by tight junction proteins such as claudins, occludins, and junctional adhesion molecules (JAMs) (Sá-Pereira et al., 2012; Keaney and Campbell, 2015).

The CNS microvasculature is formed of continuous non-fenestrated capillaries, which naturally inhibits passage of charged molecules and big particles. The endothelial cells also exhibit a comparatively significant number of mitochondria compared to other endothelial cells in the body. This huge volume of energy-producing organelles is necessary to produce the ATP needed to drive the energy-mediated transport which is normally used to carry materials past this barrier (Aird, 2007a, 2007b). The endothelial cells of the BBB have the feature of being very thin compared to other endothelial cells in the body. In addition, these cells do not express a significant number of leukocyte adhesion molecules (LAMs) as other tissues, thereby reducing the number of immune cells that can overcome the barrier to penetrate into the cerebral region (Sá-Pereira et al., 2012; Daneman, 2012).

In order to serve their duty of regulating the influx and efflux of materials to and from the brain, endothelial cells of the BBB possess two primary transporters. The first are engaged in the movement of lipophilic molecules which should usually diffuse across the cell membrane and are called efflux transporters. The second class of transporters are those engaged in moving nutrients to the CNS and waste from the CNS to the blood, called nutrient transporters at the tight junctions between these cells (Jia et al., 2013). Tight connections created by endothelial cells provide not just a physical barrier, but also constitute a molecular barrier that tightly restricts substance movement. Many research has been carried out to discover the proteins that make up the tight junction, and they include principally the occludins, claudins, and JAMs (Daneman and Prat, 2015).

Claudins are of several varieties and are expressed in diverse tissues. Research on mice models with knockout genes have helped to find claudins expressed in different organs. For example, research by Morita et al. (1999) found that mice lacking cldn5 (claudin-5) exhibited a leaky BBB for particles of sizes that ordinarily are under tight regulation. Other claudins related with the BBB are cldn3, and cldn12. Claudins 11 and 19 are also found in the nervous system, but are expressed on CNS myelin and peripheral myelin correspondingly (Nitta et al., 2003; Daneman et al., 2010). On the other hand, animals defective in occludin have a functional BBB despite the fact that occludin is highly expressed in endothelial cells of the BBB compared to other tissues. However, these occludin-deficient animals were shown to have a calcium-enriched accumulation in the brain tissues, showing that occludins play a crucial function in controlling calcium flow via the BBB (Saitou et al., 2000). For JAMs, they are a group of proteins belonging to the immunoglobulin superfamily. They are expressed in multiple tissues; however, JAM-2 and JAM-3 are specific to endothelial cells. The subtype JAM-1 is vital in maintaining the integrity of the BBB while the subtype JAM-4 was found by Daneman et al. (2010) in the BBB of mice.

The pericytes of the BBB have contractile proteins that help in restricting the breadth of blood channels in order to regulate the passage of materials via the BBB. They do this by extending lengthy cellular processes to the endothelial cells. It is however important to emphasize that these processes do not touch the endothelium physically, but do so at specialized joints called peg-and-socket junctions (Sá-Pereira et al., 2012). Also, pericytes in the BBB differ from pericytes in the body in their numbers, that is, there is a large number of pericytes located in the CNS compared to other body tissues, thereby making the ratio of pericytes to endothelial cells in the CNS higher (1:1 to 1:3) compared to other cells, such as the muscles (1:100). The embryonic origin of pericytes in tissues also comprise another difference, since CNS pericytes grow from the neural crest whereas the mesoderm is the origin of other tissue pericytes (Daneman, 2008; Daneman and Prat, 2015).

It is also vital to highlight the microglia, which are resident macrophages in the neurological system. They act as the line of defence beyond the BBB and are implicated in innate immunity. They are produced from the yolk sac and become permanently resident in the neural tissues taking on a traditional inflammatory-prone M1 phenotypic expression or the tissue repair M2 phenotypic expression (Daneman and Prat, 2015; Haruwaka et al., 2019). When the microglia show the M2 phenotype, they vigorously phagocytose apoptotic cells and clean debris from the brain region. Also, the microglia are implicated in many illnesses of the brain and contribute both as a protector of neural tissues, and also as a source of pro-inflammatory cytokines that disrupt the BBB (Thurgur and Pinteaux, 2019; Ronaldson and Davis, 2020).

Generally speaking, the neurovascular unit (NVU), including pericytes, neurons, microglia, and astrocyte end-feet, maintains the structural integrity of the barrier. The astrocytes are a form of glial cells that support neurons via the maintenance of ionic concentration and modulation of neurotransmitters (Galea, 2021). Like pericytes, they are also able to change the width of the endothelial cells based on the vascular contents needed by the brain. Studies have also revealed that astrocytes release chemicals and components that are critical in maintaining the BBB integrity (Daneman and Prat, 2015; Haruwaka et al., 2019). Particularly, the cross-talk between the endothelial cells and components of the NVU preserves the BBB integrity. Also, substances such as vascular endothelial growth factor (VEGF) and transforming growth factor-beta (TGF-*β*), released by astrocytes enhance BBB formation and maintenance (Daneman, 2008; Saunders et al., 2013; Profaci et al., 2020). Furthermore, the BBB preserves the neuronal function of the brain, supports synaptic plasticity, and prevents neurotoxicity by managing the molecular and cellular traffic between the blood and the brain.

### 4.2 Impact of systemic inflammation on BBB integrity

Inflammation is often marked by indicators, such as dolor (pain), calor (heat), rubor (redness), tumor (swelling), and functio laesa (loss of function), as well as the activation of the immune system. While the notion of inflammation invokes anxiety, it is a required physiologic reaction entrenched in mediating numerous bodily processes toward repair and homeostasis (Varatharaj and Galea, 2017; Galea, 2021). That is, regulated inflammation is good and gravitates the body toward regaining the natural physiologic condition. However, when inflammation is prolonged, it significantly affects normal physiological functioning. The same markers, factors, and cytokines necessary to drive cellular repair response can destroy cells and cause extensive damage. This extensive damage is called systemic inflammation and happens when inflammatory cascades become aggressive and attack distant tissues (Mou et al., 2022).

The cardiovascular system plays a significant part in the course of systemic inflammation development. Inflammatory cytokines released from site of injury or from infection, when sustained, perpetuates a positive feedback cascade involving the release of damage associated molecular patterns (DAMPS) from damaged cells which attracts cells that mediate innate immunity, such as, neutrophils, leukocytes, macrophages (from the bloodstream or tissue-resident macrophages) (Millán Solano et al., 2023). These cells are attracted by chemokines, mainly CCL2, CXCL1, and IL-8, which enter the bloodstream, and find their way into the systemic circulation. Also, the recruited immune cells release pro-inflammatory mediators, including TNF-*α*, IL-1β, IL-6, in a bid to clean away damaged cells and control inflammation. However, when this continues, and they are insufficient to contain the inflammation, they also continually release additional inflammatory mediators. These released mediators enter the systemic circulation in order to recruit more immune cells from different parts, a process termed the systemic inflammatory response syndrome (SIRS) (Mou et al., 2022). As this process continues, depending on the severity of inflammation, a more concerted effort is needed, over a period of time, from both the innate and acquired immune systems. For instance, lipopolysaccharide (LPS)-induced inflammation or inflammation owing to protracted ischemia–reperfusion injury can generate extensive systemic inflammation (Haruwaka et al., 2019).

This broad inflammation has the ability to harm sensitive organs, local to the site of infection/injury, or distant, and influence normal body functioning. Organs such as the kidney, lungs, liver, heart, and even the brain are not left out from this insult, potentially resulting in what is called multiple organ damage (MOD) (Cabrera et al., 2017). Naturally, the body has various mechanisms to protect delicate organs against injuries or insults from infection, and even uses mild inflammation to effect tissue repair and mediate some physiologic function. Yet, these organs are still susceptible to sustained inflammation insults, particularly those that can result in widespread systemic inflammation. The brain, being a very important organ responsible for managing the affairs of other body functions, interfaces with the body through the BBB, which as described earlier above, selectively permits materials in and out of the brain. This guarantees that the brain is preserved in a tightly regulated, immunoprivileged milieu, which is presumably secure from peripheral inflammatory insults (Munji et al., 2019; Wojciech et al., 2020; Mou et al., 2022).

However, the brain must still be aware of the state of the body so as to precisely coordinate activities that restore homeostasis. Hence, the BBB permits certain cytokines to enter the brain, through carrier-mediated transport of pro-inflammatory cytokines, which tells of the body’s inflammatory condition (Kikuchi et al., 2019). The brain is then able to launch a series of behavioral and physiologic reactions together named the sickness behavior, which include, fever, reduced appetite, weariness, social withdrawal, and disturbed sleep patterns. These responses facilitate healing and are mediated by the hypothalamic–pituitary–adrenal (HPA) axis. This axis ultimately involves the release of glucocorticoids and other stress hormones that influence immunological response (Nishioku et al., 2009; Banks et al., 2015).

During systemic inflammation, the BBB is impacted via numerous processes, such as, activation of matrix metalloproteinases (MMPs), oxidative stress and free radical damage, and direct effects of circulating cytokines on tight junction proteins. Pathways such as the NF-κB pathway, the MAPK pathway, and the JAK/STAT pathway have also been recognized by prior studies to play a role in the pathogenesis of BBB integrity disturbance (Kebir et al., 2007). The NF-κB pathway is a master regulator of the inflammatory process and is particularly crucial in inducing BBB breakdown. Pro-inflammatory cytokines (TNF-*α* or IL-1β) activate NF-κB via a sequential mechanism. The inhibitory protein IκB is first phosphorylated and destroyed, so permitting NF-κB to translocate into the nucleus of the cell. Once in the nucleus, NF-κB triggers the transcription of several inflammatory mediators, including additional cytokines, chemokines, and adhesion molecules, which spread inflammation (Rahman et al., 2018; Setiadi et al., 2019).

The MAPK pathway, comprising p38, JNK, and ERK cascades, is also activated during inflammation by cytokines and oxidative stress. Activated MAPKs phosphorylate downstream targets, resulting in the creation of inflammatory mediators and modifications in tight junction protein expression. The p38 MAPK pathway, in particular, has been shown to play a significant role in BBB breakdown. It also enhances the internalization of tight junction proteins and increases paracellular permeability (Blecharz-Lang et al., 2018; Pignolo et al., 2020). On the other hand, the JAK/STAT pathway, activated by cytokines such as IL-6 and interferons, contributes to BBB dysfunction by dimerizing STAT proteins upon activation which translocate to the nucleus, where they regulate the expression of genes involved in inflammation, cell survival, and barrier function. This route has been notably involved in the downregulation of claudin-5 and occludin expression in brain endothelial cells during inflammatory circumstances (Galea, 2021; Mou et al., 2022).

Furthermore, pro-inflammatory cytokines which are generated from immune cells, such as TNF-α and IL-1β, are raised during SIRS to encourage the recruitment of immune cells into the brain parenchyma. In normal physiologic state, the brain parenchyma is only subject to nascent immune surveillance, however during inflammation, the elevated pro-inflammatory markers induce the increased expression of adhesion molecules like intercellular adhesion molecule-1 (ICAM-1) and vascular cell adhesion molecule-1 (VCAM-1) on the endothelial cells of the BBB (Daneman and Prat, 2015; Varatharaj and Galea, 2017; Millán Solano et al., 2023). The production of these adhesion molecules helps ease the migration of neutrophils and monocytes into the brain parenchyma. Moreover, the increase in reactive oxygen species (ROS) and reactive nitrogen species (RNS) in endothelial cells due to systemic inflammation might alter claudins and occludins, which are critical structural components of the BBB. This raises the BBB’s permeability and promotes the unregulated entry of cytokines, immune cells and other inflammatory indicators, thereby altering the tightly regulated neuronal milieu (Rubin and Staddon, 1999; Obermeier et al., 2016). Concurrently, the brain responds to this by activating its own resident macrophage, the microglia. This activation initially protects the brain cells, but can aggravate inflammation by the release of more cytokines, ROS, and excitotoxic glutamate. This cascade of events can lead to neurotoxicity, synaptic malfunction, and, in severe circumstances, neuronal death (Haruwaka et al., 2019).

In ischemia–reperfusion injury (IRI), which can lead to systemic inflammation, while reperfusion is important to restore blood flow so as to prevent irreversible tissue damage, the abrupt restoration of blood flow can paradoxically trigger an inflammatory cascade. During ischemia, hypoxia-induced damage leads in the release of DAMPs, such as high-mobility group box-1 protein (HMGB1) and heat shock proteins (HSPs), which activate pattern recognition receptors (PRRs) such toll-like receptors (TLRs) on immune cells (Fernández et al., 2020; Wang et al., 2024b). This leads to the recruitment of neutrophils and the release of cytokines, increasing the inflammatory response. Reperfusion exacerbates this process by infusing oxygen, which causes a burst of ROS and exacerbates oxidative stress. These ROS not only harm cellular structures but also further impair the BBB by degrading tight junction proteins and promote endothelial death (Fernández et al., 2020).

**Figure 1:**
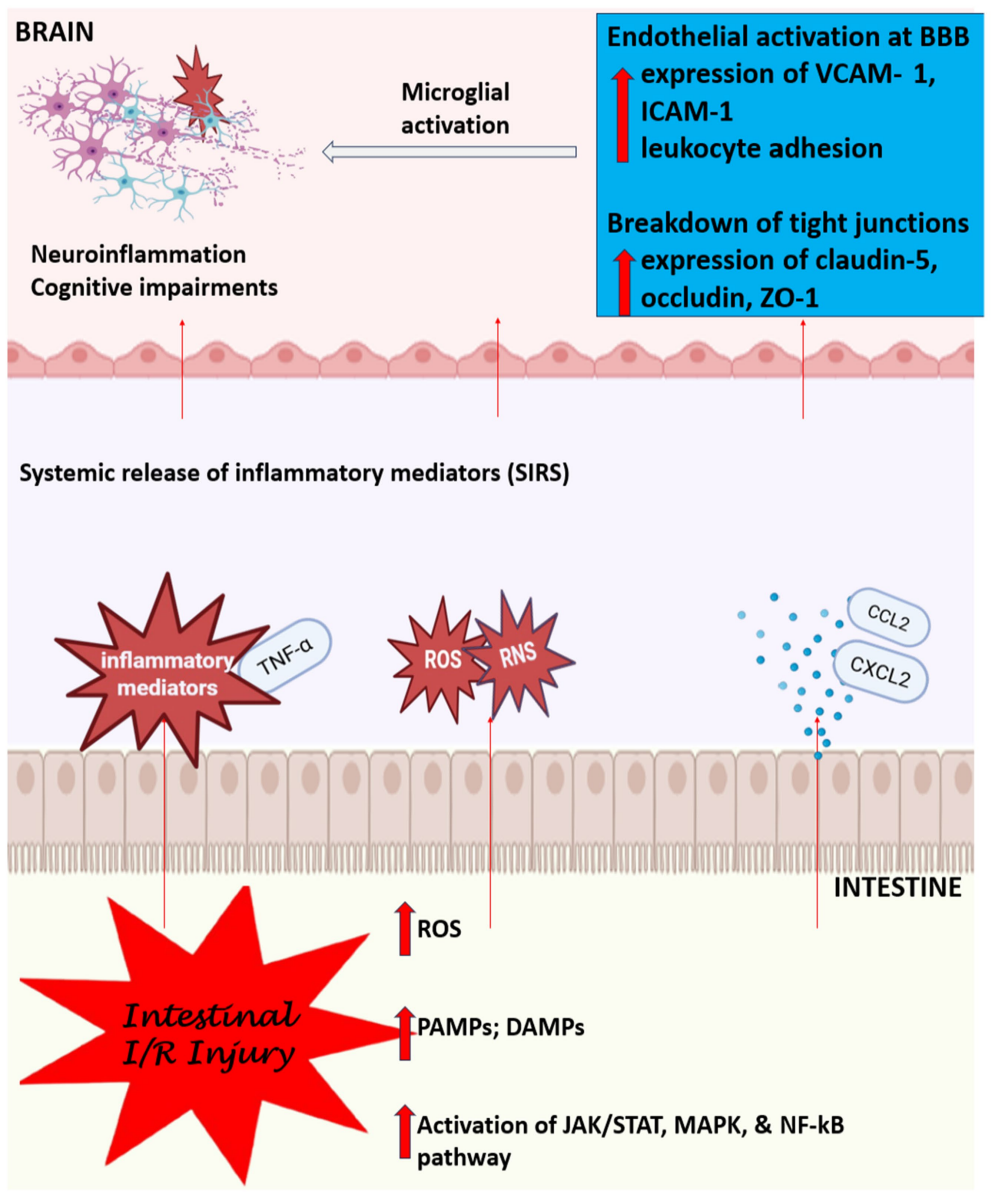
Mechanistic link between intestinal ischemia/reperfusion injury and blood–brain barrier dysfunction. The figure above shows a schematic illustration of the proposed mechanistic pathway linking intestinal ischemia/reperfusion (I/R) injury to BBB disruption via systemic inflammatory responses. Intestinal I/R triggers the release of damage-and pathogen-associated molecular patterns (DAMPs/PAMPs), reactive oxygen and nitrogen species (ROS/RNS), and proinflammatory cytokines (e.g., TNF-α). These mediators activate systemic inflammatory responses (SIRS) and signaling pathways such as JAK/STAT, MAPK, and NF-κB, leading to endothelial activation at the BBB. Upregulation of VCAM-1 and ICAM-1 promotes leukocyte adhesion, while downregulation of tight junction proteins (claudin-5, occludin, ZO-1) compromises BBB integrity. This culminates in microglial activation, neuroinflammation, and potential cognitive deficits.

The systemic inflammation caused by IRI can also influence the brain by boosting the influx of peripheral immune cells and inflammatory mediators, as earlier demonstrated, across the weakened BBB. This can lead to cerebral edema, neuroinflammation, and, in severe cases, cognitive impairments and neuronal death. Notably, the hippocampus, a region crucial for memory and learning, is particularly sensitive to these inflammatory insults, potentially linking systemic inflammation and BBB disruption to long-term cognitive deficits (Peng et al., 2019; Cowled and Fitridge, 2020; Tang et al., 2022). A summary of this mechanistic process is shown in Figure 1 below.

## 5 Cognitive dysfunction linked to intestinal I/R injury and BBB compromise

During intestinal ischemia–reperfusion (I/R) injury, the intestinal barrier integrity breaks compromised and puts in motion a cascade of events that might ultimately influence cognitive function. The gut-brain axis is implicated in this since it is the bidirectional communication network between the gastrointestinal tract and the central nervous system (Hsieh et al., 2011; Zhou et al., 2012). When intestinal I/R injury occurs, the tight junctions between intestinal epithelial cells become compromised, leading to increased intestinal permeability, a phenomenon often referred to as “leaky gut” (Liu et al., 2023). This increased permeability allows for the translocation of various substances that would normally be contained within the intestinal lumen. These substances include bacterial endotoxins (particularly lipopolysaccharide, LPS), whole bacteria, and damage-associated molecular patterns (DAMPs) released from injured intestinal cells. These translocated materials enter the portal circulation and, eventually, the systemic circulation, where they can interact with various immune cells and trigger inflammatory responses (Stewart et al., 2017). The inflammatory response initiated by these translocated materials is particularly significant since circulating inflammatory mediators can interact directly with the blood–brain barrier (BBB), leading to increased BBB permeability. This compromise in BBB integrity allows for the entry of substances that would normally be excluded from the brain parenchyma. The entry of these substances triggers a local inflammatory response within the brain tissue itself, characterized by microglial activation and the production of additional pro-inflammatory cytokines (Varatharaj and Galea, 2017; Galea, 2021; Mou et al., 2022).

In response, the brain activates the microglia which undergo morphological changes, that is, polarization to the M2 phenotype, and begin producing various inflammatory mediators, including TNF-*α*, IL-1β, and IL-6. These locally produced inflammatory mediators can directly affect neuronal function and synaptic transmission (Hinson et al., 2015). Furthermore, activated microglia release reactive oxygen species (ROS) and reactive nitrogen species (RNS), contributing to oxidative stress within the brain tissue (Wang et al., 2024c). This oxidative stress can lead to cellular damage and dysfunction, particularly in regions of the brain that are more susceptible to oxidative damage, such as the hippocampus. The hippocampus is crucial for learning and memory formation and is particularly susceptible to the effects of intestinal I/R-mediated neuroinflammation (Zlatković et al., 2014). Studies have demonstrated that hippocampal neurons are highly sensitive to the effects of systemic cytokines, which can alter synaptic plasticity, reduce long-term potentiation (LTP), and impair neurogenesis (Venkateshappa et al., 2012; Ho et al., 2015). For instance, IL-1β disrupts LTP by modulating NMDA receptor activity (Lynch, 2015; Prieto et al., 2019), while TNF-*α* induces excitotoxicity through its effects on glutamate homeostasis (Boraso, 2012). Furthermore, sustained inflammation can lead to structural changes within the hippocampus, including reduced dendritic spine density, alterations in synaptic protein expression and changes in neuronal connectivity patterns (Perry, 2010).

The hippocampus is not the only brain region affected by intestinal IRI-mediated neuroinflammation. Other areas involved in cognition such as the amygdala, prefrontal cortex can also be affected (Block et al., 2005; Zhou et al., 2012). The progression of cognitive dysfunction following intestinal I/R injury follows a temporal pattern in which acute effects may manifest as attention deficits and mild memory impairment; and as the inflammatory response persists, more substantial cognitive deficits, such as impaired spatial memory formation, reduced cognitive flexibility, decreased processing speed, altered emotional regulation, and compromised learning ability, may emerge (Guan et al., 2009; Dou et al., 2019).

### 5.1 Clinical evidence and animal studies on cognitive impairment

Studies have provided evidence for the link between gut-originated inflammation and cognitive decline (Rudzki and Maes, 2020; Jia et al., 2024). Conditions such as inflammatory bowel disease (IBD), sepsis, and chronic gut dysbiosis, which are characterized by systemic inflammation, have been associated with impairments in memory, attention, and executive function. For instance, Attree et al. (2003) and Kennedy et al. (2014) reported that patients with IBD exhibited significant deficits in cognitive performance compared to healthy controls, which correlated with elevated systemic levels of pro-inflammatory cytokines, particularly IL-6 and TNF-*α*. Similarly, patients with sepsis often develop sepsis-associated encephalopathy (SAE), a condition characterized by altered mental status and long-term cognitive impairments. In these patients, the translocation of gut-derived endotoxins, such as lipopolysaccharides (LPS), into systemic circulation is a recognized contributor to systemic inflammation and subsequent BBB compromise (Gofton and Young, 2012). Elevated inflammatory markers in these patients correlate with neuroinflammatory changes, microglial activation, and neuronal damage in the hippocampus and prefrontal cortex, leading to persistent cognitive deficits (Sonneville et al., 2023). Moreover, emerging evidence suggests that gut-originated inflammation may play a role in neurodegenerative diseases such as Alzheimer’s disease (AD). The gut-brain axis and systemic inflammation have been implicated in the deposition of amyloid-beta plaques and tau pathology in the brain, both hallmarks of AD (Jia et al., 2024). These findings indicate that chronic inflammation originating from the gut can influence brain pathology and cognitive outcomes.

Animal models have also helped in elucidating the mechanisms linking intestinal I/R injury to cognitive impairment. Experimental studies involving rodents subjected to intestinal I/R injury consistently demonstrate systemic inflammatory responses that impact brain function. For example, Yang et al. (2020a) observed that intestinal I/R injury in rats induced significant cognitive deficits, as evidenced by impairments in spatial memory tasks. These deficits were associated with elevated levels of IL-1β, TNF-*α*, and ROS in the hippocampus, as well as structural changes such as reduced synaptic density and neuronal apoptosis. Studies further confirmed that the disruption of the intestinal barrier during I/R injury facilitated the translocation of LPS, which exacerbated systemic inflammation and compromised BBB integrity (Song et al., 2021; Li et al., 2022).

Similarly, a study by Zhou et al. (2012) demonstrated that intestinal I/R injury in mice resulted in hippocampal microglial activation and upregulation of pro-inflammatory cytokines. This neuroinflammatory response was accompanied by reduced expression of tight junction proteins in the BBB, suggesting that systemic inflammation originating from the gut directly impacts BBB permeability and brain inflammation. The resulting cognitive deficits were attributed to disruptions in hippocampal plasticity, as evidenced by altered long-term potentiation and reduced neurogenesis. Furthermore, research on the impact of gut-originated inflammation on behavior and cognition revealed that rats subjected to intestinal I/R injury exhibited anxiety-like behavior and deficits in recognition memory, which were linked to elevated circulating levels of IL-6 and TNF-α. Histological analysis revealed neuronal degeneration and oxidative stress in the prefrontal cortex and hippocampus, providing a mechanistic basis for the observed cognitive impairments (Holmes et al., 2020; Singh et al., 2024). It is important to note that direct clinical activity remains relatively limited and this gap underscores the need for translational research.

The table below summarizes some of the available treatment options that have shown potential in experimental models and their clinical status (Table 1).

**Table 1:** Clinical trials for intestinal ischemia reperfusion injury.

Strategy	Clinical trial status	Key findings/limitations	References
Anesthetics	No clinical trials for I/R	Protective in animal studies; clinical relevance unclear	Hou et al. (2023) and Huang et al. (2024)
Antioxidants	No clinical trials for I/R	Preclinical efficacy; translation needed	Li and Zheng (2021) and Wang et al. (2021)
Ischemic preconditioning	No controlled human trials	Promising in animals; human data limited to other organs	Mallick et al. (2004) and Hu et al. (2022)
Wnt pathway modulators	No clinical trials for I/R	Mechanistic promise; not yet in clinical use	Zhang et al. (2024a)

## 6 Mechanisms of intestinal-brain signaling

The gut-brain axis is a bidirectional communication system linking the gastrointestinal (GI) tract and the central nervous system (CNS), which plays a pivotal role in maintaining homeostasis across many physiological processes (Arneth, 2018; Lu et al., 2024). Disruptions in this axis, such as those induced by ischemia–reperfusion (I/R) injury in the intestine, have been implicated in a range of pathological outcomes, including cognitive dysfunction (Yang et al., 2020b).

### 6.1 The gut-brain axis and its role in signaling during I/R injury

The gut-brain axis is a complex, bidirectional communication network involving neural, hormonal, and immune pathways. Under normal conditions, this axis helps regulate functions such as digestion, immunity, and mood (Powell et al., 2017; Arneth, 2018). During intestinal I/R injury, this communication can become dysregulated, leading to systemic inflammation, altered neural signaling, and, ultimately, cognitive dysfunction (Zhang et al., 2024c).

#### 6.1.1 Neuroimmune pathways

Intestinal I/R injury induces ischemic damage to the gut, leading to the activation of immune responses within the gut mucosa. These immune responses, including the release of pro-inflammatory cytokines (e.g., TNF-α, IL-1β), can signal to the brain via the vagus nerve and the systemic circulation (Ma et al., 2020). This results in the activation of microglia (resident immune cells in the brain) and the subsequent neuroinflammatory cascade in the CNS, which has been shown to contribute to cognitive deficits (Nuszkiewicz et al., 2024).

#### 6.1.2 Gut-derived factors

Intestinal I/R injury leads to the leakage of gut-derived factors such as bacterial endotoxins (lipopolysaccharide, LPS), metabolites, and cytokines into the bloodstream (Nie et al., 2024). These factors can cross the blood–brain barrier (BBB), further triggering neuroinflammation in the brain. LPS, in particular, has been shown to increase the permeability of the BBB, allowing immune cells and inflammatory mediators to infiltrate the brain, exacerbating cognitive dysfunction (Takata et al., 2021).

#### 6.1.3 Vagus nerve signaling

The vagus nerve, a key player in the gut-brain axis, is involved in the regulation of inflammation. During I/R injury, the vagus nerve can relay signals from the inflamed gut to the brain, which can result in the modulation of both peripheral and central inflammatory pathways (Bosi, 2021). Vagal nerve stimulation has been shown to have anti-inflammatory effects, which may be leveraged therapeutically to prevent or ameliorate cognitive impairment following I/R injury (Timmers et al., 2020).

### 6.2 Role of microbiota in influencing cognitive outcomes

The gut microbiota, which consists of trillions of microorganisms residing in the gastrointestinal tract, plays an essential role in regulating intestinal homeostasis and influencing brain function through the gut-brain axis (Ding et al., 2020). Recent studies have highlighted the importance of gut microbiota in shaping the outcomes of I/R injury and its potential impact on cognitive function (Kobayashi et al., 2021).

#### 6.2.1 Dysbiosis and neuroinflammation

I/R injury induces significant changes in the composition of the gut microbiota, a condition known as dysbiosis. Dysbiosis can lead to an overgrowth of pathogenic bacteria and a reduction in beneficial microbes such as Lactobacillus and Bifidobacterium (Wang et al., 2013). This imbalance contributes to the increased permeability of the gut barrier, allowing the translocation of harmful bacterial products (such as lipopolysaccharides (LPS)) into the bloodstream, where they can activate the brain’s immune system and promote neuroinflammation, which is a major contributor to cognitive dysfunction (Guerville and Boudry, 2016; Liu et al., 2023).

#### 6.2.2 Short-chain fatty acids (SCFAs)

SCFAs, including butyrate, propionate, and acetate, are produced by gut microbiota during the fermentation of dietary fibers. These metabolites have been shown to exert neuroprotective effects by maintaining the integrity of the blood–brain barrier (BBB), modulating immune responses, and promoting neuronal health (Wang et al., 2018). I/R injury-induced dysbiosis can result in reduced SCFA production, contributing to increased neuroinflammation and BBB breakdown. This highlights the therapeutic potential of modulating the microbiota to prevent or mitigate cognitive dysfunction post-I/R injury (Zhang et al., 2024a).

#### 6.2.3 Gut-brain metabolites

In addition to SCFAs, gut microbiota also produces metabolites such as tryptophan, which serves as a precursor for serotonin, a neurotransmitter involved in mood regulation and cognitive function. The altered microbial profile during I/R injury can influence the synthesis of these critical metabolites, leading to changes in brain chemistry that may underlie cognitive impairment (Sun et al., 2016).

### 6.3 Neural and hormonal pathways involved in gut-brain communication

The communication between the gut and brain involves complex neuronal and hormonal networks that might be disturbed following I/R injury.

#### 6.3.1 Neural pathways

As mentioned earlier, the vagus nerve is the primary neural pathway involved in gut-brain signaling. It transmits afferent signals from the gut to the brain and has been implicated in modulating inflammation and cognitive function (Wang et al., 2024c). Vagal nerve stimulation has shown promise in attenuating neuroinflammation and improving cognitive outcomes following systemic injuries such as I/R (Wang et al., 2024d). Additionally, the enteric nervous system (ENS), often referred to as the “second brain,” can also influence brain function via local signaling mechanisms. The ENS communicates with the brain through neuropeptides such as substance P and vasoactive intestinal peptide, which are involved in modulating inflammatory responses (Marsilio, 2019).

#### 6.3.2 Hormonal pathways

Gut hormones, such as ghrelin, leptin, and cortisol, play a key role in regulating appetite, metabolism, and stress responses. After I/R injury, dysregulation of these hormones can further impact brain function (Jiao and Luo, 2022). For example, increased levels of cortisol due to stress responses can exacerbate neuroinflammation and cognitive decline. On the other hand, ghrelin, which has neuroprotective properties, may be altered by I/R injury, contributing to cognitive dysfunction. Understanding the interplay of these hormonal signals during I/R injury is crucial for developing targeted therapeutic strategies (Ma et al., 2022).

### 6.4 Potential therapeutic targets in the gut-brain axis to prevent cognitive dysfunction

Given the substantial significance of the gut-brain axis in influencing cognitive function during I/R injury, treatment techniques that target this axis show tremendous promise. Several possible therapeutic targets have been identified:

#### 6.4.1 Microbiota modulation

Probiotics, prebiotics, and synbiotics are being explored as potential therapeutic approaches to restore gut microbiota balance in the context of I/R injury (Menafra et al., 2024). These interventions can help reduce intestinal inflammation, promote the production of beneficial metabolites (e.g., SCFAs), and improve BBB integrity. Clinical studies have shown that probiotics can attenuate neuroinflammation and improve cognitive outcomes in animal models of I/R injury (Rahmati et al., 2019; Thakkar et al., 2023).

#### 6.4.2 Vagus nerve stimulation (VNS)

VNS has been shown to have anti-inflammatory effects by modulating the cholinergic anti-inflammatory pathway (Lei and Duan, 2021). Clinical and preclinical studies suggest that VNS can reduce neuroinflammation and protect against cognitive decline in various injury models, including those involving I/R. VNS may represent a promising approach to mitigate cognitive dysfunction following intestinal I/R injury (Du et al., 2024).

#### 6.4.3 Anti-inflammatory agents

Targeting the inflammatory cascade induced by I/R injury is a key strategy in preventing cognitive dysfunction (Tang et al., 2022). Nonsteroidal anti-inflammatory drugs (NSAIDs), corticosteroids, and more recently developed anti-cytokine therapies (e.g., TNF-*α* inhibitors) could help reduce systemic and neuroinflammation (Kim and Lee, 2024).

#### 6.4.4 SCFA supplementation

SCFA supplementation is another potential therapeutic avenue. By restoring the production of SCFAs, it may be possible to improve gut barrier function, reduce systemic inflammation, and preserve BBB integrity. Studies exploring the use of SCFA supplements in neuroinflammation and cognitive disorders are ongoing (Shi et al., 2020; Guo et al., 2025).

#### 6.4.5 Neuroprotective agents

Pharmacological agents that promote neuronal survival and protect against neuroinflammation, such as antioxidants or specific enzyme inhibitors (e.g., COX-2 inhibitors), may also hold therapeutic potential (Choi et al., 2010). These agents could be used in combination with gut-targeted therapies to provide a more comprehensive approach to mitigating cognitive dysfunction post-I/R (Liu et al., 2023).

## 7 Therapeutic approaches to mitigate BBB dysfunction and cognitive decline

Intestinal ischemia–reperfusion (I/R) injury and the resulting systemic inflammation can severely compromise the blood–brain barrier (BBB), leading to cognitive dysfunction (Zhang et al., 2024b). In response to this, therapeutic strategies have been explored to mitigate BBB dysfunction and prevent cognitive decline. Among these, anti-inflammatory interventions are central, as inflammation plays a key role in exacerbating BBB permeability and neurological impairments (Cervellati et al., 2020).

### 7.1 Anti-inflammatory therapies to lower systemic inflammation

Systemic inflammation is a critical mediator in the pathophysiology of BBB disruption and cognitive dysfunction following intestinal I/R injury (Wei et al., 2021). Inflammatory mediators, including cytokines, chemokines, and reactive oxygen species (ROS), are released during I/R and travel to the brain, where they can directly damage the BBB and promote neuroinflammation (Song et al., 2020). Therapeutic approaches that target systemic inflammation hold great promise in protecting the BBB and mitigating cognitive decline (Yu et al., 2024).

#### 7.1.1 Use of cytokine inhibitors

Cytokines, particularly pro-inflammatory cytokines such as tumor necrosis factor-alpha (TNF-*α*), interleukin (IL)-1β, and IL-6, are known to contribute significantly to BBB dysfunction and neuroinflammation following I/R injury (Jurcau and Simion, 2021). Elevated cytokine levels increase endothelial cell permeability, leading to leakage of plasma proteins and immune cells across the BBB (Jurcau and Simion, 2021). Additionally, cytokines can activate microglial cells, which in turn release more inflammatory mediators, exacerbating neuroinflammation and contributing to neuronal injury (Smith et al., 2012; Huang et al., 2021).

To combat this, cytokine inhibitors, which target specific inflammatory pathways, are being investigated for their potential to reduce BBB permeability and improve cognitive outcomes in models of intestinal I/R injury (Ronaldson and Davis, 2020). These inhibitors can be classified into two broad categories: monoclonal antibodies and small-molecule inhibitors.

##### 7.1.1.1 Monoclonal antibodies

Antibodies targeting specific cytokines, such as TNF-α inhibitors (e.g., infliximab and adalimumab), have been shown to decrease the levels of systemic inflammation and reduce BBB dysfunction (Esposito and Cuzzocrea, 2009). In preclinical models, TNF-α inhibitors have been demonstrated to reduce BBB leakage and mitigate neuronal damage (Gu et al., 2021). These therapies are also being studied in clinical settings for conditions like inflammatory bowel disease (IBD) and sepsis, where I/R injury may exacerbate systemic inflammation and impair BBB integrity (Mehrzadi et al., 2023).

##### 7.1.1.2 Small-molecule inhibitors

Small-molecule inhibitors that block cytokine signaling pathways, such as the Janus kinase (JAK) inhibitors, have shown promise in reducing inflammation in I/R models (Panda et al., 2024). JAK inhibitors block the signaling cascade downstream of cytokine receptors, preventing the activation of pro-inflammatory genes (Malemud and Pearlman, 2009). For example, drugs like quercetin, which target the JAK–STAT signaling pathway, have demonstrated the ability to reduce systemic inflammation and potentially prevent BBB breakdown, although further studies are needed to assess their cognitive benefits in the context of intestinal I/R injury (Zalpoor et al., 2022).

#### 7.1.2 Dietary treatments (e.g., probiotics, prebiotics)

Dietary interventions that modulate the gut microbiota have gained increasing attention for their potential to mitigate systemic inflammation, protect the BBB, and reduce cognitive decline (Shi et al., 2020). Intestinal microbiota plays a critical role in regulating immune responses and systemic inflammation, influencing both local (intestinal) and remote (brain) inflammatory processes (Banfi et al., 2021). Dysbiosis, or an imbalance in the gut microbiome, has been associated with increased systemic inflammation and exacerbated BBB dysfunction in a variety of conditions, including I/R injury (Kaur et al., 2023).

##### 7.1.2.1 Probiotics

Probiotics are live microorganisms that, when consumed in adequate amounts, provide health benefits by restoring the balance of gut microbiota (Sánchez et al., 2017). Probiotic strains, such as Lactobacillus and Bifidobacterium, have been shown to reduce intestinal permeability and modulate systemic inflammation (Corridoni et al., 2012). In models of intestinal I/R injury, probiotics can reduce the levels of circulating pro-inflammatory cytokines, such as TNF-α and IL-6, and promote the expression of anti-inflammatory cytokines, like IL-10 (Virk et al., 2024). Additionally, probiotics can enhance intestinal barrier function by promoting the expression of tight junction proteins and reducing oxidative stress. In terms of BBB protection, probiotics may reduce microglial activation, a key contributor to neuroinflammation and cognitive decline (Mou et al., 2022). Several studies suggest that probiotic supplementation can significantly reduce BBB disruption, although the exact mechanisms through which probiotics achieve this are still under investigation (Dziedzic and Saluk, 2022; Peterson, 2020; Soltanian et al., 2025).

##### 7.1.2.2 Prebiotics

Prebiotics are non-digestible food components, typically fibers, that selectively stimulate the growth and activity of beneficial gut microbiota. Prebiotic consumption has been shown to modify the gut microbiome and promote an anti-inflammatory profile by enhancing the abundance of beneficial bacteria, such as Firmicutes and Bacteroidetes (Bilal et al., 2022). Prebiotics can also reduce the levels of harmful bacteria and the production of their associated inflammatory metabolites (Zhang et al., 2024c). In the situation of I/R injury, prebiotics may reduce systemic inflammation, thereby alleviating neuroinflammation and protecting the BBB (Zhang et al., 2024a). In addition to probiotics and prebiotics, other dietary interventions, including polyphenols, omega-3 fatty acids, and other anti-inflammatory nutrients, are also under investigation for their potential to regulate gut inflammation and protect the BBB (Winiarska-Mieczan et al., 2023). Studies have shown that the consumption of omega-3 fatty acids has been shown to reduce microglial activation and promote neuroprotection in models of neuroinflammation (Zendedel et al., 2015; Chen et al., 2017; Chen et al., 2018).

### 7.2 Pharmacological treatments to restore BBB integrity

Pharmacological interventions designed to restore BBB integrity after I/R injury are essential to reduce the risk of cognitive decline associated with BBB disruption. Two promising approaches include the use of tight junction modulators and antioxidant therapies. Both strategies aim to restore the structural and functional properties of the BBB and prevent the cascade of inflammatory and oxidative processes that can contribute to neurodegeneration.

#### 7.2.1 Tight connection modulators

Tight junctions are specialized structures between endothelial cells that form a selective barrier, restricting the passage of solutes and maintaining the integrity of the BBB. In the context of I/R injury, disruption of tight junction proteins, such as claudins, occludin, and zonula occludens (ZO) proteins, leads to increased BBB permeability (Shi et al., 2016). This disruption is often a result of the release of inflammatory cytokines, oxidative stress, and matrix metalloproteinase (MMP) activation. Restoring tight junction integrity is thus a promising strategy for protecting the BBB and preventing cognitive dysfunction (Lochhead et al., 2020).

Several pharmacological treatments are being studied to modify tight junctions and restore BBB integrity.

##### 7.2.1.1 Melatonin

Melatonin, a neurohormone that regulates circadian rhythms, has been shown to have protective effects on the BBB in models of I/R injury (Zhang et al., 2024b). Studies indicate that melatonin can preserve the integrity of tight junctions by reducing oxidative stress and suppressing the expression of pro-inflammatory cytokines (Qin et al., 2019; Mehrzadi et al., 2023). It has been demonstrated to upregulate the expression of tight junction proteins (e.g., ZO-1, occludin) and prevent their degradation following I/R injury. By preventing tight junction disruption, melatonin helps maintain BBB permeability and protects against neuroinflammation, thereby mitigating cognitive decline. The ability of melatonin to cross the BBB and act directly on the brain makes it a particularly attractive candidate for treating BBB dysfunction (Chen et al., 2022).

##### 7.2.1.2 Statins

Statins, commonly used for lowering cholesterol, have also shown potential in protecting BBB integrity. Statins exert their effects through the inhibition of HMG-CoA reductase, an enzyme involved in cholesterol biosynthesis, but they also have anti-inflammatory and neuroprotective properties (Saeedi Saravi et al., 2017). Preclinical studies suggest that statins can reduce BBB permeability by enhancing the expression of tight junction proteins and decreasing the activity of MMPs, which are implicated in the degradation of tight junctions (Liu et al., 2021). Statins may also modulate endothelial nitric oxide (NO) production, improving endothelial function and preserving BBB integrity. In models of ischemic injury, statins have been shown to attenuate BBB disruption and reduce the inflammatory response, which may help prevent cognitive dysfunction (Morandi et al., 2011).

##### 7.2.1.3 Angiotensin II type 1 receptor (AT1R) blockers

Angiotensin II is a vasoconstrictor that plays a role in the regulation of blood pressure, but it also contributes to BBB disruption during ischemic events. AT1R blockers, such as losartan, have been investigated for their potential to mitigate BBB damage (Huang and Zhang, 2024). AT1R blockers reduce the inflammatory response and oxidative stress induced by angiotensin II, which can lead to improved tight junction protein expression and BBB integrity (Huang and Zhang, 2024). Studies have shown that these blockers can reduce BBB leakage and improve cognitive outcomes in animal models of I/R injury, suggesting their potential as a therapeutic strategy to prevent BBB dysfunction and cognitive decline (Xiong et al., 2020; Gelosa et al., 2022).

#### 7.2.2 Antioxidant therapy

Oxidative stress, which arises from an imbalance between reactive oxygen species (ROS) production and the antioxidant defense mechanisms, is a key contributor to BBB dysfunction and neuroinflammation following intestinal I/R injury (Song et al., 2020). ROS damage endothelial cells, disrupt tight junctions, and activate inflammatory pathways, all of which contribute to BBB permeability. Therefore, antioxidant therapies aimed at reducing oxidative stress represent a promising approach for protecting the BBB and mitigating cognitive decline (Rochfort et al., 2014).

Several antioxidant therapies have showed potential in preserving the BBB in the context of I/R injury.

##### 7.2.2.1 N-acetylcysteine (NAC)

NAC is a well-known antioxidant that serves as a precursor to glutathione, a major cellular antioxidant. By replenishing glutathione levels, NAC helps to neutralize ROS and reduce oxidative stress (Aldini et al., 2018). In models of intestinal I/R injury, NAC has been shown to reduce oxidative damage to endothelial cells, preserve tight junction integrity, and protect against BBB leakage. In addition to its antioxidant effects, NAC also has anti-inflammatory properties, further enhancing its potential as a therapeutic strategy for BBB protection and cognitive preservation (Keshk et al., 2020; Ikram et al., 2024).

##### 7.2.2.2 Curcumin

Curcumin, the active compound found in turmeric, has been widely studied for its anti-inflammatory and antioxidant properties. Curcumin can reduce ROS production, inhibit the activation of nuclear factor kappa-light-chain-enhancer of activated B cells (NF-κB), and modulate the expression of pro-inflammatory cytokines (Hammed et al., 2025). Studies have demonstrated that curcumin can protect the BBB by reducing oxidative stress and preserving tight junction protein expression (Sarada et al., 2015; Zhang et al., 2017). In animal models of I/R injury, curcumin has been shown to attenuate BBB breakdown and improve cognitive function, suggesting its potential as a neuroprotective agent in BBB-related disorders (Wang et al., 2019; Wu et al., 2021).

Mitochondrial-targeted antioxidants: Mitochondria are a major source of ROS, and mitochondrial dysfunction plays a critical role in oxidative stress and BBB disruption (Bhat et al., 2015). Mitochondrial-targeted antioxidants, such as MitoQ, have been developed to selectively target mitochondria and reduce ROS generation at the cellular level. MitoQ has been shown to reduce oxidative stress and improve mitochondrial function in endothelial cells, thereby preserving tight junction integrity and preventing BBB leakage (Zinovkin et al., 2023; Fields et al., 2023). In preclinical models, MitoQ has demonstrated efficacy in reducing cognitive decline associated with I/R injury, indicating its potential for therapeutic use in BBB dysfunction (Ibrahim et al., 2023; Pang et al., 2024).

##### 7.2.2.3 Vitamin E

Vitamin E, a fat-soluble antioxidant, has been extensively studied for its ability to neutralize lipid peroxides and protect cell membranes from oxidative damage (Orucha et al., 2011). In the case of BBB dysfunction, vitamin E has been shown to reduce oxidative stress, preserve endothelial cell integrity, and enhance the expression of tight junction proteins. Supplementation with vitamin E has been shown to attenuate BBB leakage and protect against cognitive impairment following I/R injury, suggesting its potential as a protective agent for the BBB (Freeman and Keller, 2012; Orellana-Urzúa et al., 2023).

### 7.3 Role of gut microbiota regulation and probiotics

The gut microbiota, composed of trillions of microorganisms, plays a critical role in maintaining intestinal homeostasis, regulating immune responses, and influencing systemic inflammation (Pickard et al., 2017). Dysbiosis, an imbalance in the gut microbiota, has been implicated in the pathogenesis of various diseases, including those involving neuroinflammation and BBB dysfunction (Sochocka et al., 2019).

Recent studies have highlighted the potential of modulating the gut microbiota, either through dietary interventions or probiotic supplementation, to restore microbiota balance and reduce the inflammatory response, which could ultimately protect the BBB and improve cognitive function (Dahiya and Nigam, 2023; Fock and Parnova, 2023).

#### 7.3.1 Gut microbiota and its impact on BBB integrity

The gut microbiota can influence BBB integrity via several mechanisms, including the modulation of the immune system and the production of metabolites that affect the brain. During I/R injury, gut-derived endotoxins such as lipopolysaccharide (LPS) can translocate from the gut into the systemic circulation, triggering inflammation and immune activation (Suganya and Koo, 2020; Nie et al., 2024). These endotoxins have been shown to increase the permeability of the BBB, allowing for the passage of inflammatory cytokines and immune cells into the brain (Jaeger et al., 2009). In addition to LPS, gut-derived metabolites such as short-chain fatty acids (SCFAs) can influence BBB function, with certain SCFAs promoting anti-inflammatory pathways that protect the integrity of the BBB (Ahmed et al., 2022).

The gut microbiota also modulates the immune response by interacting with immune cells in the gut-associated lymphoid tissue (GALT) (Nie et al., 2024). Dysbiosis can lead to the activation of pro-inflammatory pathways, such as the NF-κB pathway, which in turn increases the production of cytokines and inflammatory mediators that compromise the BBB (Sanz and De Palma, 2009). Importantly, a balanced gut microbiota supports the production of anti-inflammatory cytokines, such as IL-10, which help protect both the intestinal barrier and the BBB (Mou et al., 2022).

Thus, maintaining a healthy and balanced gut microbiota may prevent the systemic inflammation that causes BBB malfunction and cognitive impairment following intestinal I/R injury (Zhang et al., 2024c).

#### 7.3.2 Probiotics and their effects on the gut-brain axis

Probiotics, defined as live microorganisms that confer health benefits when administered in adequate amounts, have gained attention as potential therapeutic agents for modulating gut microbiota and improving outcomes in diseases associated with systemic inflammation and BBB dysfunction (de LeBlanc and LeBlanc, 2014). Probiotics can restore the balance of the gut microbiota, reduce intestinal permeability, and suppress systemic inflammation, thereby indirectly protecting the BBB and the brain from the effects of I/R injury (Zhou et al., 2023).

Several methods have been hypothesized for how probiotics regulate the gut-brain axis and protect the BBB.

##### 7.3.2.1 Restoration of gut barrier integrity

Probiotics have been shown to enhance the function of tight junction proteins, such as occludin and zonula occludens (ZO-1), which are crucial for maintaining intestinal epithelial integrity (Serek and Oleksy-Wawrzyniak, 2021). By improving intestinal barrier function, probiotics reduce the translocation of harmful substances (e.g., LPS) into the bloodstream, thereby decreasing systemic inflammation and preventing BBB disruption (Maguire and Maguire, 2019).

##### 7.3.2.2 Modulation of immune responses

Probiotics exert immunomodulatory effects by influencing both innate and adaptive immune responses. They can enhance the production of anti-inflammatory cytokines like IL-10 and reduce the production of pro-inflammatory cytokines such as TNF-*α* and IL-6 (Cristofori et al., 2021). This anti-inflammatory effect helps attenuate the inflammatory cascade that leads to BBB dysfunction. In addition, probiotics can regulate the activity of immune cells, including T cells and macrophages, contributing to a balanced immune response that protects the BBB (Rastogi and Singh, 2022).

##### 7.3.2.3 Reduction of oxidative stress

Probiotics also play a role in modulating oxidative stress, which is a key contributor to BBB breakdown. Some probiotic strains, such as Lactobacillus and *Bifidobacterium*, have been shown to reduce the production of reactive oxygen species (ROS) and enhance the activity of antioxidant enzymes (Yang et al., 2020a; Zheng et al., 2023). By reducing oxidative damage, probiotics help preserve the integrity of the endothelial cells that form the BBB, thereby preventing neuroinflammation and cognitive decline (Martínez-Guardado et al., 2022).

##### 7.3.2.4 Short-chain fatty acid production

Certain probiotics produce short-chain fatty acids (SCFAs) such as acetate, propionate, and butyrate, which are by-products of the fermentation of dietary fiber by gut bacteria. SCFAs are known to have anti-inflammatory properties and can influence the function of the BBB (Cuciniello et al., 2023). For example, butyrate has been shown to strengthen the tight junctions of endothelial cells, reduce oxidative stress, and suppress neuroinflammation. By promoting the production of SCFAs, probiotics may contribute to BBB protection and cognitive preservation (Welcome, 2019).

#### 7.3.3 Probiotic strains and their effects in I/R injury models

Several research have studied the effects of particular probiotic strains in models of intestinal I/R injury and their capacity to protect the BBB and retain cognitive function.

##### 7.3.3.1 Lactobacillus species

Strains such as *Lactobacillus rhamnosus* and *Lactobacillus plantarum* have demonstrated beneficial effects in attenuating systemic inflammation and improving gut barrier function (Zhao et al., 2020). In animal models of I/R injury, Lactobacillus strains have been shown to reduce the levels of pro-inflammatory cytokines, such as TNF-α and IL-1β, and prevent BBB disruption (Li et al., 2022). Additionally, these probiotics have been found to reduce oxidative stress and prevent cognitive deficits following I/R injury (Sun et al., 2016).

##### 7.3.3.2 Bifidobacterium species

*Bifidobacterium breve* and *Bifidobacterium longum* have also been studied for their potential to restore gut microbiota balance and protect the BBB (Valvaikar et al., 2024). In animal models, Bifidobacterium supplementation has been shown to improve gut permeability, reduce inflammation, and enhance the expression of tight junction proteins in both the gut and the BBB. These effects contribute to the preservation of cognitive function in models of I/R injury (Dong and Mayer, 2024).

##### 7.3.3.3 Saccharomyces boulardii

This probiotic yeast has been shown to have protective effects on both the intestinal and BBB integrity in I/R injury models. *S. boulardii* helps restore gut barrier function, reduce systemic inflammation, and protect against cognitive decline by modulating the gut-brain axis and reducing oxidative stress (Babaei et al., 2024).

#### 7.3.4 Clinical implications and problems

The potential of probiotics as a therapeutic strategy for mitigating BBB dysfunction and cognitive decline is supported by promising preclinical findings, but clinical evidence remains limited (Sproten et al., 2024). While some small-scale clinical studies have reported beneficial effects of probiotics on cognitive function and gut health in various conditions, more robust, large-scale clinical trials are needed to validate the efficacy of probiotics specifically for preventing BBB disruption and cognitive impairment following I/R injury (Ghassab et al., 2024).

Challenges in translating these findings to clinical practice include the selection of appropriate probiotic strains, determining the optimal dosage, and understanding the complex interactions between the gut microbiota and the brain. Furthermore, while probiotics appear to be generally safe, their effects can be strain-specific, and long-term use or potential interactions with other medications require careful consideration.

The Table 2 below summarizes the therapeutic strategies in use for mitigating intestinal I/R-induced gut-brain axis dysfunction.

**Table 2:** Therapeutic strategies for intestinal I/R injury.

Mechanism	Therapeutic strategy	Effect
Oxidative stress	Antioxidants (e.g., natural polyphenols, melatonin, vitamin D agonists) (Yang et al., 2020b; Kim et al., 2022)	Suppression of ROS production and NF-κB activation reduce BBB permeability
	Agents targeting NADPH oxidase and NOS (Dumitrescu et al., 2018; Chen et al., 2024; Wang et al., 2024a)	Reduced ROS-mediated phosphorylation of tight junction proteins
Gut dysbiosis	Probiotics, prebiotics, fecal microbiota transplantation (FMT) (Chen et al., 2022; Chidambaram et al., 2022)	Restoration of intestinal microbial balance and SCFA production, improvement in gut barrier integrity
	Antibiotic treatment (with caution) (Kalogeris et al., 2017)	Experimental depletion of gut bacteria reduces systemic inflammation and brain injury in animal models, although with mixed results
Inflammatory signaling (NF-κB, TLR4, NLRP3)	Anti-inflammatory agents (e.g., NF-κB inhibitors, specific cytokine blockers such as anti-TNF therapies) (Akhtar and Choudhry, 2011; Kim et al., 2022)	Inhibition of proinflammatory cytokine production and restoration of tight junction protein expression.
Gene regulation (miRNAs; epigenetics)	miRNA inhibitors (targeting miR-21-5p), HDAC inhibitors (e.g., butyrate supplementation) (Chidambaram et al., 2022; Hu et al., 2022)	Modulation of gene expression to maintain barrier integrity and reduce inflammation.
Ischemic preconditioning	Exposes the tissue to short bouts of I/R injury, thereby making the tissue more resistant to the effects of I/R injury (Mallick et al., 2004; Wong et al., 2021)	Enhances tissue tolerance to I/R

### 7.4 Neuroprotective strategies for cognitive function

Neuroprotection is the use of interventions that protect the nervous system from injury or degeneration. In the context of BBB dysfunction and cognitive decline due to intestinal I/R injury, neuroprotective strategies aim to enhance neuronal survival, reduce neuroinflammation, and restore brain function (Jain and Jain, 2019). Neurotrophic factors and pharmacological agents targeting neuroinflammation represent two promising approaches for protecting cognitive function and promoting recovery following BBB compromise (Sulhan et al., 2020).

#### 7.4.1 Use of neurotrophic factors

Neurotrophic factors are proteins that support the growth, survival, and differentiation of neurons. These factors play a critical role in neuronal plasticity, which is vital for memory formation and cognitive function (Li et al., 2022). Under pathological conditions, such as those associated with intestinal I/R injury, the expression and activity of neurotrophic factors are often reduced. Restoring or enhancing neurotrophic signaling may help counteract the detrimental effects of BBB disruption and promote cognitive recovery (Miyake et al., 2020).

Several neurotrophic agents have been found to preserve neurons, increase BBB integrity, and decrease cognitive impairment following I/R injury.

##### 7.4.1.1 Brain-derived neurotrophic factor (BDNF)

BDNF is one of the most well-studied neurotrophic factors in the central nervous system (CNS). It supports neuronal survival, synaptic plasticity, and cognitive function (Failla et al., 2016). In animal models of I/R injury, BDNF has been shown to reduce neuroinflammation, protect neurons from apoptosis, and preserve cognitive function (Asadi et al., 2018). BDNF exerts its effects by binding to the TrkB receptor, activating downstream signaling pathways such as the PI3K/Akt and MAPK/ERK pathways, which promote cell survival and synaptic plasticity (Ma et al., 2013). Moreover, BDNF has been shown to enhance the expression of tight junction proteins in endothelial cells, thereby supporting BBB integrity. Increasing BDNF expression or mimicking its activity could serve as a therapeutic strategy to counteract the cognitive deficits associated with I/R-induced BBB disruption (Hernandez et al., 2022; Yang et al., 2022).

##### 7.4.1.2 Glial cell-derived neurotrophic factor (GDNF)

GDNF is another neurotrophic factor that plays a key role in neuronal survival, particularly in dopaminergic neurons. GDNF has shown promise in protecting the brain from ischemic injury by promoting neurogenesis, reducing neuroinflammation, and enhancing BBB integrity (Singh et al., 2023). Studies have demonstrated that GDNF administration following I/R injury can reduce neuronal damage, restore BBB function, and improve cognitive performance (Korley et al., 2016; Appunni et al., 2021). Additionally, GDNF has been shown to reduce the expression of pro-inflammatory cytokines in the brain, suggesting its potential as a therapeutic target for mitigating neuroinflammation and cognitive decline (Mokhtari et al., 2017; Delgado-Minjares et al., 2021).

##### 7.4.1.3 Vascular endothelial growth factor (VEGF)

VEGF is a critical regulator of angiogenesis and vascular permeability. In the framework of I/R injury, VEGF can promote BBB repair and protect against vascular damage (Hoseinzadeh et al., 2022). While excessive VEGF expression can lead to increased BBB permeability, controlled VEGF signaling has been shown to support the survival and function of endothelial cells that make up the BBB (Simons et al., 2016). In animal models of ischemic stroke, VEGF has been shown to improve neuronal survival, reduce infarct size, and enhance cognitive function. VEGF may also promote neuroprotection indirectly by enhancing the clearance of neurotoxic metabolites and reducing oxidative stress (Kim et al., 2019).

##### 7.4.1.4 Insulin-like growth factor-1 (IGF-1)

IGF-1 is a potent neurotrophic factor that is involved in the regulation of neuronal growth and survival. IGF-1 has been shown to have neuroprotective effects in various neurological conditions, including I/R injury (Gong et al., 2021). In preclinical models, IGF-1 has been found to reduce the inflammatory response, protect neurons from oxidative damage, and improve cognitive outcomes following I/R injury. IGF-1 signaling also plays a role in maintaining BBB integrity by promoting the expression of tight junction proteins and inhibiting the breakdown of the extracellular matrix (Freese et al., 2017).

#### 7.4.2 Pharmacological agents for neuroinflammation

Neuroinflammation, characterized by the activation of microglia, astrocytes, and the release of pro-inflammatory cytokines, is a major contributor to cognitive dysfunction and BBB breakdown following intestinal I/R injury (Ronaldson and Davis, 2020). Targeting neuroinflammation with pharmacological agents is a promising strategy to reduce the systemic inflammatory response, preserve BBB integrity, and prevent cognitive decline (Fu et al., 2018). Several classes of pharmacological agents have been investigated for their ability to modulate neuroinflammation and provide neuroprotection:

##### 7.4.2.1 Nonsteroidal anti-inflammatory drugs (NSAIDs)

NSAIDs, such as ibuprofen and aspirin, are commonly used to reduce inflammation and pain. In the situation of I/R injury, NSAIDs have been shown to attenuate the production of pro-inflammatory cytokines (e.g., TNF-*α*, IL-6) and reduce microglial activation, which are key drivers of neuroinflammation (Ajmone-Cat et al., 2010). Although NSAIDs have demonstrated efficacy in reducing neuroinflammation and improving BBB function, their use is limited by potential side effects, including gastrointestinal bleeding and cardiovascular risks (Kaduševičius, 2021). Therefore, alternative agents with more selective anti-inflammatory effects are being explored.

##### 7.4.2.2 Corticosteroids

Corticosteroids, such as dexamethasone, are potent anti-inflammatory agents that have been shown to reduce microglial activation and prevent neuroinflammation following I/R injury (Soiberman et al., 2017). These agents can also reduce the expression of adhesion molecules, thereby limiting the infiltration of immune cells into the brain and preserving BBB integrity (Salvador et al., 2014). However, long-term use of corticosteroids is associated with adverse effects, including immune suppression and increased susceptibility to infections, which limits their clinical utility (Brahmer et al., 2018). The development of corticosteroid analogs with fewer side effects could provide a more targeted approach to managing neuroinflammation (Kalra et al., 2022).

##### 7.4.2.3 Minocycline

Minocycline, an antibiotic with anti-inflammatory properties, has gained attention as a potential neuroprotective agent in models of ischemic injury (Sharma et al., 2010). Minocycline has been shown to inhibit microglial activation, reduce the release of pro-inflammatory cytokines, and protect neurons from oxidative damage (Abdo Qaid et al., 2024). Studies have demonstrated that minocycline can reduce neuroinflammation, prevent BBB disruption, and improve cognitive outcomes following I/R injury. Minocycline is currently being explored in clinical trials as a potential treatment for neuroinflammatory diseases such as Alzheimer’s and Parkinson’s disease (Zhao et al., 2023).

##### 7.4.2.4 Nicotinamide adenine dinucleotide (NAD+) precursors

NAD + is a coenzyme involved in cellular energy metabolism and is critical for maintaining neuronal function. NAD + levels decline with age and under pathological conditions, such as I/R injury, leading to increased neuroinflammation and neuronal damage (Narne and Phanithi, 2023). Administration of NAD + precursors, such as nicotinamide riboside (NR), has been shown to reduce microglial activation, enhance mitochondrial function, and promote neuronal survival (Alegre and Pastore, 2023). Preclinical studies have demonstrated that NAD + precursors can attenuate neuroinflammation and improve cognitive function in models of I/R injury, suggesting their potential as a therapeutic strategy for neuroprotection (Khoury et al., 2018).

##### 7.4.2.5 Selective inhibitors of pro-inflammatory cytokines

Targeting specific pro-inflammatory cytokines, such as TNF-α, IL-1β, and IL-6, has been explored as a therapeutic approach to mitigate neuroinflammation and cognitive decline (Brod, 2022). Monoclonal antibodies or small molecule inhibitors that specifically block the activity of these cytokines have shown promise in preclinical studies (Hanke et al., 2016). For example, TNF-α inhibitors, such as infliximab, have been shown to reduce neuroinflammation and improve cognitive function in animal models of ischemic injury. However, the potential for adverse effects and the complexity of cytokine signaling pathways require careful consideration in clinical application (Turner et al., 2014; Zhao et al., 2021).

## 8 Limitations and future directions

The evidence that links intestinal I/R damage to cognitive dysfunction keeps mounting and various experimental models have proved this. A major limitation, however, lies in the fact that many of the previous research relies primarily on animal models that do not fully recapitulate the complexity, heterogeneity, and comorbidities of human intestinal I/R injury, thereby limiting the predictive value for patient outcomes. While studies like those of Attree et al. (2003) and Kennedy et al. (2014) reveal a link between intestinal dysfunction and cognitive dysfunction, they are often limited by their small sample sizes, heterogeneous patient populations, and insufficient control for confounding factors such as comorbidities, medication, nutritional status, and psychological stress. This makes such studies largely observational or cross-sectional. Also, as at the moment of writing, no randomized controlled trials (RCTs) have been conducted to specifically assess whether attenuating intestinal inflammation or barrier dysfunction improves cognitive outcomes in post-IIRI settings or related clinical conditions.

To bridge this knowledge gap, future studies would need to focus on developing and validating already established clinically relevant animal models on large animals that better reflect human disease and allow for the study of long-term outcomes and tissue repair to set the stage for future human trials (Gonzalez et al., 2015; McKinney-Aguirre et al., 2024). Another direction could be the development of humanized animal models such as mice colonized with human microbiota or with humanized immune systems (Ka et al., 2023). Integration of multi-omics approaches, which encompasses genomics, transcriptomics, proteomics, and metabolomics, in future studies of IIRI-related cognitive decline will help reveal interrelated regulatory networks and identify actionable molecular targets. To progress the body of knowledge concerning IIRI-related cognitive dysfunction and translate findings to the bedside, there’s need for interdisciplinary efforts.

## 9 Conclusion

Intestinal ischemia–reperfusion (I/R) injury affects the brain through systemic inflammation, gut microbiota dysbiosis, oxidative stress, and vagus nerve activation, leading to BBB disruption and cognitive impairment. Future research should explore the specific roles of gut microbes, long-term effects on the gut-brain axis, and vagus nerve involvement, with a focus on personalized probiotic interventions. Clinically, restoring gut microbiota balance, reducing inflammation, and mitigating oxidative stress may improve cognitive outcomes, but clinical trials are needed. Recognizing the gut’s role in neurological health highlights the importance of multi-organ communication in disease prevention and treatment.
